# Minor Non-Disabling Stroke Patients with Large Vessel Severe Stenosis or Occlusion Might Benefit from Thrombolysis

**DOI:** 10.3390/brainsci11070945

**Published:** 2021-07-19

**Authors:** Wansi Zhong, Ying Zhou, Kemeng Zhang, Shenqiang Yan, Jianzhong Sun, Min Lou

**Affiliations:** 1 Department of Neurology, The Second Affiliated Hospital of Zhejiang University, School of Medicine, Hangzhou 310009, China; 21718233@zju.edu.cn (W.Z.); zh_ying@zju.edu.cn (Y.Z.); 3140104539@zju.edu.cn (K.Z.); shenqiangyan@zju.edu.cn (S.Y.); 2 Department of Radiology, The Second Affiliated Hospital of Zhejiang University, School of Medicine, Hangzhou 310009, China; 2191009@zju.edu.cn

**Keywords:** minor non-disabling stroke, stenosis, alteplase, excellent outcome

## Abstract

Background: The benefit of alteplase in minor non-disabling acute ischemic stroke (AIS) is unknown. We aimed to explore the clinical efficacy of alteplase-treatment in minor non-disabling stroke in clinical practice. Methods: We used a prospectively collected database of AIS patients who were being assessed for thrombolysis with alteplase. Minor non-disabling AIS was identified as patients with baseline National Institutes of Health Stroke Scale (NIHSS) score ≤ 5 and a score 0 or 1 on each baseline NIHSS score item (items 1a to 1c being 0). Results: A total of 461 patients with minor non-disabling AIS were included and among them 240 (52.1%) patients were treated with alteplase and 113 (24.5%) patients had severe stenosis/occlusion of large vessels. No significant association of 90-day excellent outcome was found with alteplase-treatment (77.1% vs. 80.5%, *p* 1 = 0.425; OR 0.911, 95% CI 0.428 to 1.940; *p* 2 = 0.808). However, among patients with severe stenosis/occlusion of large vessels, alteplase-treatment was independently associated with excellent outcome (74.4% vs. 45.7%, *p* 1 = 0.005; OR 4.709, 95% CI 1.391 to 11.962; *p* 2 = 0.010). Conclusion: Although alteplase-treatment did not result in an excellent outcome in general minor non-disabling stroke patients, it may work in those specific patients who had severe stenosis/occlusion of large vessels.

## 1. Introduction

A recent study reported that more than one-half of acute ischemic stroke (AIS) hospitalizations had mild deficits, accounting for four of every 10 patients who received intravenous thrombolysis (IVT) therapy in national US practice [1]. Despite only initial mild neurological symptoms, nearly a third of patients with minor stroke not receiving alteplase treatment suffered functional disability at 90-day post stroke [2,3,4]. The 2019 guidelines for AIS thus recommended that minor disabling AIS patients could be treated with alteplase [5]. Treatment with alteplase in minor stroke has increased in recent years to reduce potential post-stroke disability [6,7].

However, minor non-disabling stroke still brings difficulties for the decision-making of thrombolysis in AIS [8]. A clinical trial failed to demonstrate the benefit of alteplase among patients with minor non-disabling AIS [9]. However, in that trial, the number of recruited patients (n = 313) was much less than expectation, leading to the uncertainty of the result. Moreover, the definition of minor non-disabling AIS remains elusive. Evidence on the necessity of IVT for minor non-disabling AIS patients is still ambiguous.

Intracranial arterial stenosis was demonstrated to be associated with an increased risk of stroke and disability in minor stroke [10]. The infarct progression in patients with severe stenosis/occlusion of large vessels is one of the main causes of neurological deterioration in AIS patients [11], while a considerable number of these patients initially present mild symptoms. Thus, we aimed to explore the clinical efficacy of alteplase-treatment in minor non-disabling stroke and further investigated whether the presence of stenosis/occlusion of large vessels would influence the clinical effects of alteplase-treatment in those patients.

## 2. Materials and Methods

### 2.1. Patients

We reviewed our prospectively collected database CIPPIS (Clinicaltrials.gov ID: NCT03367286) for AIS patients who were admitted in our hospital between 2009 and 2019. Patients were enrolled who (1) had a diagnosis of minor non-disabling AIS; (2) underwent CT or MR angiography within 4.5 h of symptom onset. All patients were clinically eligible for thrombolysis according to local guidelines and clinical judgment of physician. Patients were excluded who (1) had modified Rankin Scale (mRS) ≥ 2 prior to the index stroke; (2) had received mechanical thrombectomy; (3) had poor image quality; (4) were lost at follow-up at 90-days. Minor non-disabling AIS patients were identified as patients with baseline National Institutes of Health Stroke Scale (NIHSS) score ≤5 and a score 0 or 1 on each baseline NIHSS score item (items 1a to 1c being 0) [12]. Alteplase administration in minor non-disabling AIS patients was totally left at the physician’s discretion. The study was approved by the local human ethics committee (the Ethics Committee of the Second Affiliated Hospital, School of Medicine, Zhejiang University: EC-20190801-1059). Written informed consent was obtained from patients or relatives.

### 2.2. Imaging Protocol

MR was performed on a 3.0-T system (Sigma Excite HD, General Electric, Milwaukee, WI, USA). Foam pads were inserted into the space between the patient’s head and the MR head coil to minimize head motion. Sequences included diffusion weighed imaging (repetition time = 4000 ms; echo time = 69.3 ms; b-value = 1000 s/mm^2^; slice thickness = 5.0 mm; interslice gap = 1.0 mm) and time-of-flight MR angiography (repetition time = 20 ms; echo time = 3.2 ms; flip angle = 15; slice thickness = 1.4 mm, three slabs).

CT perfusion was performed on a dual-source 64-slice CT scanner (SOMATOM Definition Flash; Siemens, Forchheim, Germany), including non-contrast CT head scan (120 kV, 320 mA, contiguous 5 mm axial slices), and volume perfusion CT (100 mm in the *z*-axis, 4 s delay after start of contrast medium injection, 74.5 s total imaging duration, 80 kV, 120 mA, slice thickness 1.5 mm, collimation 32 × 1.2 mm). A 60-mL bolus of contrast medium (Iopamidol; Braccosine, Shanghai, China) was used at a flow rate of 6 mL/s, followed by a 20 mL saline chaser at 6 mL/s. CT angiography images were reconstructed from volume perfusion CT in axial, coronal, and sagittal planes with 20-mm-thick maximum intensity projection.

### 2.3. Image Analysis and Classification of Patients

Based on baseline MR or CT angiography, the stenotic degree of symptomatic intracranial vessels (including intracranial segment of internal carotid artery, M1 or M2 segment of middle cerebral artery, A1 or A2 segment of anterior cerebral artery, P1 or P2 segment of posterior cerebral artery, and basilar artery) was graded as normal (stenosis = 0), mild to moderate (0 < stenosis < 70%), severe (70 ≤ stenosis < 100%) and occlusive (stenosis = 100%) [11]. Two neurologists independently reviewed the data blind of clinical data, with rater discrepancies settled by consensus discussion. Hemorrhagic transformation was identified on brain CT or MRI scan sat 24 h after treatment. Symptomatic intracranial hemorrhage (sICH) was defined as hemorrhagic transformation associated with an increase of ≥4 points on the NIHSS score from baseline assessment, according to the European Cooperative Acute Stroke Study (ECASS) II trial [13].

The mRS scores were followed up by telephone questionnaire at 90-days after symptom onset by a third party who were blinded to the patients’ clinical data. The telephone questionnaire had been validated and used in previous trials [14,15,16]. Excellent outcome was defined as mRS score 0–1 at 90-day follow-up.

### 2.4. Statistical Analysis

All metric and normally distributed variables were reported as mean ± standard deviation; non-normally distributed variables as median (25th–75th percentile). Categorical variables were presented as frequency (percentage). Comparison between groups were assessed by using Student’s t test for data that followed normal distribution, Mann–Whitney U test for data that did not follow normal distribution, and Fisher’s Exact test for categorical data. Binary logistic regression analysis was used to evaluate clinical outcome and generate odds ratios. Variables with a *p* value of <0.1 in univariate analysis were included in the multivariate analysis. A *p* value of <0.05 was considered to be statistically significant. Statistical analysis was performed using SPSS 26.0 (SPSS, Inc, Chicago, IL, USA) and R 4.0.1(the R Foundation for Statistical Computing, Vienna, Austria).

## 3. Results

Of 1857 consecutive AIS patients, 578 were minor non-disabling and clinically eligible for thrombolysis. Finally, 461 patients were included in the analysis, after 117 patients were excluded due to mRS ≥ 2 prior to the index stroke (n = 19), receiving mechanical thrombectomy (n = 8), losing follow-up at 90-day (n = 90).

Of the included patients, mean age was 69 ± 13 years and 152 (33.0%) were female, median baseline NIHSS score was 2 (1–3), median onset to door time (ODT) was 122 (77–186) min, and 240 (52.1%) patients were treated with alteplase. Among the entire cohort, 348 (75.5%) patients had normal or mild to moderate stenosis of large vessels, and 113 (24.5%) patients had severe stenosis/occlusion of large vessels.

Table 1 shows that alteplase-treated patients had higher baseline NIHSS scores (3 vs. 1, *p* < 0.001), higher rate of large vessel severe stenosis/occlusion (32.5% vs. 15.8%, *p* < 0.001) and hemorrhagic transformation (14.3% vs. 3.2%, *p* < 0.001). Six (2.5%) patients developed sICH in alteplase-treated patients. No significant differences in other variables were observed. As Table 2 shows, patients with excellent outcome were younger and had lower baseline NIHSS score, higher rate of smoking, and lower rate of symptomatic intracranial hemorrhage at 24 h (all *p* < 0.05).

In the entire population, the rate of 90-day excellent outcome was similar between patients treated with alteplase and those not treated with alteplase (77.1% vs. 80.5%, *p* 1 = 0.425; OR 0.911, 95% CI 0.428 to 1.940; *p* 2 = 0.808). In patients with severe stenosis/occlusion of large vessels (n = 113), comparison of clinical characteristics between alteplase-treated and untreated groups is presented in Table S1. Alteplase-treated patients had higher baseline NIHSS scores (3 vs. 2, *p* < 0.001), lower rate of diabetes (19.2% vs. 40.0%, *p* = 0.034), higher rate of smoking (37.2% vs. 15.6%, *p* = 0.040), and hemorrhagic transformation at 24 h (17.9% vs. 0, *p* = 0.039). The rate of 90-day excellent outcome was significantly higher in patients treated with alteplase than in those not treated with alteplase (74.4% vs. 45.7%, *p* 1 *=* 0.005; OR 4.709, 95% CI 1.391 to 11.962; *p* 2 = 0.010) after adjusting for age, onset to door time, baseline NIHSS score, smoking, and atrial fibrillation. The power calculation was 90.6%. Among patients with normal vessels or mild to moderate stenosis of large vessels (n = 348), alteplase-treatment was not associated with excellent outcome (78.4% vs. 87.1%, *p* 1 = 0.033; OR 0.708, 95% CI 0.347 to 1.445; *p* 2 = 0.343). (Table 3).

## 4. Discussion

Our study did not find significant difference in excellent outcome among minor non-disabling AIS patients treated and untreated with alteplase. However, minor non-disabling AIS patients with severe stenosis/occlusion of large vessels could benefit from alteplase-treatment.

In the entire population with minor non-disabling AIS, our result was consistent with the PRISMS trial which demonstrated no increase of the likelihood of excellent functional outcome after thrombolysis [9]. Data from a nationwide multicentric database in China also demonstrated that no significant advantage of alteplase-treatment over dual antiplatelet therapy or aspirin was found in minor stroke with NIHSS score of 0–3 [17]. Although the definition of minor non-disabling stroke is difficult, these current data do not support routinely considering alteplase-treatment in unselected minor non-disabling stroke.

Further analysis from our data demonstrated that minor non-disabling stroke with severe stenosis/occlusion could benefit from alteplase-treatment. Neurological deterioration commonly occurred in patients with severe stenosis of large vessels [11,18,19] It was reported that patients with mild deficits (NIHSS ≤ 5) and large vessel occlusion deteriorated more often in non-thrombolyzed patients than thrombolyzed patients within 3 months after onset [20]. Meanwhile, the NIHSS score was unable to exactly reflect the presence of intracranial artery occlusion. Our data shows that 24.5% of minor non-disabling stroke patients existed with severe stenosis/occlusion. Thus, our finding indicates that AIS patients who have large vessel severe stenosis or occlusion need to be treated even if they only present minor non-disabling symptoms. Of note, thrombolysis was found to be a potential effective treatment for those with large vessel severe stenosis/occlusion. The clot lysis due to alteplase may prevent the original thrombus extension, and even infarct progression due to non-recanalization. Future prospective studies are needed to confirm our results and the exact mechanisms.

The risk of sICH in minor stroke patients receiving alteplase-treatment varied from 0% to 3.7% [21,22,23]. As reported, the sICH rate of minor stroke in the TIMS-China and CNSR-III registry was 2.1% despite a baseline NIHSS score lower than 3 [17]. In the present analysis, six patients (2.5%) developed sICH in the minor non-disabling stroke patients treated with alteplase. Although the rate of sICH in our study was not comparable to previous studies because of a different definition of minor stroke, alteplase-treatment seemed not to increase the risk of sICH.

With the increased rate of reperfusion therapy, angiography imaging is widely applied during the hyper-acute stage of AIS. Our finding suggested that the selection of eligible patients based on the degree of large artery stenosis might potentially help the decision-making for thrombolysis in minor non-disabling AIS patients. A recent study also found that screening for large-vessel occlusion with CT angiography in patients with acute minor stroke is cost-effective and associated with improved outcomes [24]. The utility of early CT angiographic detection of stenosis/occlusion for minor non-disabling stroke could be recommended, especially in the comprehensive stroke center where emergent assessment of intra-cranial and extra-cranial vessels is indispensable [25,26].

Additionally, evidence on the necessity of endovascular therapy (EVT) for minor non-disabling AIS patients with large vessel occlusion is lacking and ambiguous. The evidence of EVT for AIS patients with large vessel stenosis was not strong according to 2019 Guidelines, which state that “The usefulness of emergent or urgent carotid endarterectomy/carotid angioplasty and stenting when clinical indicators or brain imaging suggests a small infarct core with large territory at risk (e.g., penumbra), compromised by inadequate flow from a critical carotid stenosis or occlusion (IIb, B-NR)” [5]. To date, the evidence from clinical trials is lacking to support the EVT in AIS patients with minor stroke. In practice, minor non-disabling AIS and their relatives may be hesitant to make decisions whether to receive EVT due to its risk and high costs. Our results, that demonstrated the benefit of IVT in minor non-disabling AIS patients with severe stenosis or occlusion, provided a good choice for minor non-disabling stroke with severe stenosis or occlusion and suggested the benefit of reperfusion therapy.

There are several limitations in our study. First, the study is retrospectively analyzed, though the database was prospectively collected. Currently, it is only hypothesis-generating and future prospective studies are needed. Second, the definition of “not clearly disabling” was subjective, although efforts were made for the consistency assessment of clinicians. Third, not all deficits are captured by the NIHSS. The severity of posterior circulation and right hemispheric symptoms are underrepresented on the stroke scale yet may still lead to poor outcomes. The identification of minor non-disabling stroke still needs to be refined in the future. Fourth, our sample size was modest and was performed at a single center; confirmation and extension of the study in larger and multi-center cohorts is needed. Finally, since patients who received EVT were excluded in this study, we did not analyze the efficiency of MT in minor non-disabling ischemic stroke patients, which needs further study in the future.

## 5. Conclusions

Our study does not support alteplase-treatment in unselected patients with minor non-disabling stroke. However, our results do suggest that alteplase-treatment may be beneficial in minor non-disabling stroke with severe stenosis/occlusion of large vessels. Randomized trials are needed to confirm these findings.

## Figures and Tables

**Table 1:** Univariate comparison of characteristics in alteplase-treated and untreated groups in patients with minor non-disabling acute ischemic stroke.

Variables	Alteplase-Treated (n = 240)	Untreated (n = 221)	*p* Value
Age, years, ±SD	68 ± 12	70 ± 14	0.099
Female (%)	79 (32.9)	73 (33.0)	1.000
NIHSS (IQR)	3 (2–3)	1 (1–2)	<0.001
ODT, min (IQR)	126.5 (76–173)	120 (83.5–203.5)	0.060
**Risk factors**			
Hypertension (%)	167 (67.6)	148 (67.0)	0.550
Diabetes (%)	52 (21.7)	60 (27.1)	0.192
Prior stroke or TIA (%)	41 (17.1)	48 (21.7)	0.238
Atrial fibrillation (%)	51 (21.3)	35 (15.8)	0.152
Smoking (%)	94 (39.2)	61 (31.0)	0.088
**Imaging data**			
Large vessel severe stenosis or occlusion (%)	78 (32.5)	35 (15.8)	<0.001
Site of vessel stenosis or occlusion (%)			0.250
ICA	12 (10.9)	4 (5.7)	
MCA	80 (72.7)	51 (72.9)	
BA	5 (4.5)	1 (1.5)	
PCA	13 (11.8)	13 (18.6)	
ACA	0 (0)	1 (1.4)	
Hemorrhagic transformation at 24 h, %	34/238 (14.3)	5/155 (3.2)	<0.001
Symptomatic intracranial hemorrhage at 24 h, %	6/238 (2.5)	0/155 (0)	0.085

NIHSS, National Institutes of Health Stroke Scale score; ODT, Onset to Door Time; TIA, Transient Ischemic Attack; ICA, Internal Carotid Artery; MCA, Middle Cerebral Artery; BA, Basilar Artery; PCA, Posterior Cerebral Artery; ACA, Anterior Cerebral Artery.

**Table 2:** Univariate comparison of characteristics in patients with minor non-disabling acute ischemic stroke according to neurological outcome.

Variables	mRS 0–1 (n = 363)	mRS 2–6 (n = 98)	*p* Value
Age, years, ±SD	67 ± 13	74 ± 11	<0.001
Female (%)	115 (31.7)	37 (37.8)	0.277
NIHSS (IQR)	2 (1–3)	3 (2–4)	<0.001
ODT, min (IQR)	120.0 (77.0–187.0)	143.5 (89.8–180.8)	0.558
**Risk factors**			
Hypertension (%)	242 (66.7)	73 (74.5)	0.145
Diabetes (%)	83 (22.9)	29 (29.6)	0.185
Prior stroke or TIA (%)	66 (18.2)	23 (23.5)	0.250
Atrial fibrillation (%)	61 (16.8)	25 (25.5)	0.058
Smoking (%)	131 (37.9)	24 (26.4)	0.049
**Imaging data**			
Large vessel severe stenosis or occlusion (%)	74 (20.4)	39 (39.8)	<0.001
Site of vessel stenosis or occlusion (%)			0.773
ICA	9 (7.1)	7 (13.0)	
MCA	94 (74.6)	37 (68.5)	
BA	4 (3.2)	2 (3.7)	
PCA	18 (14.3)	8 (14.8)	
ACA	1 (0.8)	0 (0)	
Hemorrhagic transformation at 24 h, %	26/311 (8.4)	13/82 (15.9)	0.060
Symptomatic intracranial hemorrhage at 24 h, %	1/311 (0.3)	5/82 (6.1)	0.002

mRS, modified Rankin Scale; NIHSS, National Institutes of Health Stroke Scale score; ODT, Onset to Door Time; TIA, Transient Ischemic Attack; ICA, Internal Carotid Artery; MCA, Middle Cerebral Artery; BA, Basilar Artery; PCA, Posterior Cerebral Artery; ACA, Anterior Cerebral Artery.

**Table 3:** Univariate comparison and binary logistic regression analysis for neurological outcome.

	Variables	mRS 0–1	mRS 2–6	*p* 1 Value	*p* 2 Value
Entire cohort (n = 461)	Alteplase-Treated (%)	185 (77.1)	55 (22.9)	0.425	0.808
	Untreated (%)	178 (80.5)	43 (19.5)		
Patients with severe stenosis or occlusion (n = 113)	Alteplase-Treated (%)	58 (74.4)	20 (25.6)	0.005	0.010
	Untreated (%)	16 (45.7)	19 (54.3)		
Patients without severe stenosis/occlusion (n = 348)	Alteplase-Treated (%)	127 (78.4)	35 (21.6)	0.033	0.343
	Untreated (%)	162 (87.1)	24 (12.9)		

mRS, modified Rankin Scale. *p* 1: the *p* value of univariate analysis; *p* 2: the *p* value of binary logistic regression analysis.

## Data Availability

The data that support the findings of this study can be made available from the corresponding author on reasonable request.

## Supplementary Materials

The following are available online at https://www.mdpi.com/article/10.3390/brainsci11070945/s1, Table S1: Univariate comparison of characteristics in alteplase-treated and untreated groups in minor non-disabling acute ischemic stroke patients with severe stenosis/occlusion of large vessels.
