# Supplementary material for: Minor Non-Disabling Stroke Patients with Large Vessel Severe Stenosis or Occlusion Might Benefit from Thrombolysis
Source: Brain Sci. 2021 Jul 19;11(7):945. doi: 10.3390/brainsci11070945 (PMC8306755; doi:10.3390/brainsci11070945)
Supplement: Supplementary file 1 [file brainsci-11-00945-s001.zip › brainsci-1252607-supplementary.pdf]

Supplemental Table S1. Univariate comparison of characteristics in alteplase-treated and untreated groups in minor non-disabling acute ischemic stroke patients with severe stenosis/occlusion of large vessels.

Variables	Alteplase-Treated (n=78)	Untreated (n=35)	*P* value
Age, years, ±SD	70±13	73±15	0.251
Female (%)	29 (37.2)	14 (40.0)	0.835
NIHSS (IQR)	3 (2-3)	2 (1-2)	<0.001
ODT, min (IQR)	127.0 (78.8-193.5)	120 (60.0-192.0)	0.700
**Risk factors**			
Hypertension (%)	56 (71.8)	26 (74.3)	0.824
Diabetes (%)	15 (19.2)	14 (40.0)	0.034
Prior stroke or TIA (%)	18 (23.1)	10 (28.6)	0.638
Atrial fibrillation (%)	18 (23.1)	10 (28.6)	0.638
Smoking (%)	29 (37.2)	5 (15.6)	0.040
**Imaging data**			
Site of vessel stenosis or occlusion (%)			0.620
ICA	12 (15.4)	4 (11.4)	
MCA	56 (71.8)	26 (74.3)	
BA	3 (3.8)	0 (0)	
PCA	7 (9.0)	5 (14.3)	
ACA	0 (0)	0 (0)	
Hemorrhagic transformation at 24 h, %	14 / 78 (17.9)	0 / 26 (0)	0.019
Symptomatic intracranial hemorrhage at 24 h, %	4 / 78 (5.1)	0 /26 (0)	0.570

NIHSS, National Institutes of Health Stroke Scale score; ODT, Onset to Door Time; TIA, Transient Ischemic Attack; ICA, Internal Carotid Artery; MCA, Middle Cerebral Artery; BA, Basilar Artery; PCA, Posterior Cerebral Artery; ACA, Anterior Cerebral Artery.
